# Cardiac and vascular phenotypes in the apolipoprotein E-deficient mouse

**DOI:** 10.1186/1423-0127-19-22

**Published:** 2012-02-13

**Authors:** Elisardo C Vasquez, Veronica A Peotta, Agata L Gava, Thiago MC Pereira, Silvana S Meyrelles

**Affiliations:** 1 Department of Physiological Sciences, Health Sciences Center, Federal University of Espirito Santo, Vitoria, ES, Brazil; 2 Emescam School of Health Sciences, Vitoria, ES, Brazil; 3 The University of Iowa, Iowa City, IA, USA; 4 Biotechnology Graduate Program, Health Sciences Center, Federal University of Espirito Santo, Vitoria, ES, Brazil; 5 Federal Institute of Education, Science and Technology (IFES), Vila Velha, ES, Brazil

**Keywords:** Hypercholesterolemia, atherosclerosis, mouse, cardiac function, vascular senescence

## Abstract

Cardiovascular death is frequently associated with atherosclerosis, a chronic multifactorial disease and a leading cause of death worldwide. Genetically engineered mouse models have proven useful for the study of the mechanisms underlying cardiovascular diseases. The apolipoprotein E-deficient mouse has been the most widely used animal model of atherosclerosis because it rapidly develops severe hypercholesterolemia and spontaneous atherosclerotic lesions similar to those observed in humans. In this review, we provide an overview of the cardiac and vascular phenotypes and discuss the interplay among nitric oxide, reactive oxygen species, aging and diet in the impairment of cardiovascular function in this mouse model.

## Introduction

Over the last two decades, the mouse has become a very important experimental animal in studies of physiology and a growing number of pathophysiologies due to its easy breeding, short generation time and the availability of inbred strains. The limitations of the small size of the mouse for studying cardiac and vascular hemodynamics and function have been overcome in our and other laboratories due to advances in surgical techniques, as in Borst et al. [1], who described detailed procedures for the analysis of cardiac functional parameters in the mouse.

As a consequence of the progressive advancement of molecular biology techniques, it is possible to knockout and restore endogenous genes or add exogenous genes into the mouse, allowing the development of mouse models for human diseases. Two decades ago, the first gene-targeted murine model of atherosclerosis was created by the inactivation of the apolipoprotein E (apoE) gene by homologous recombination [2,3]. Among the genetically engineered models, the apoE-deficient (apoE^-/-^) mouse is considered to be one of the most relevant models because it develops spontaneous hypercholesterolemia and arterial lesions similar to those observed in humans.

In the apoE^-/- ^mouse, most of the plasma cholesterol (PC) is found in the atherogenic lipoprotein fractions, namely the very-low-density lipoprotein (VLDL), intermediate-density lipoprotein (IDL) and low-density lipoprotein (LDL) fractions, and this profile is aggravated by a Western-type diet. On a chow diet, apoE^-/- ^mice exhibit increased total PC (~8-fold), triglycerides (1.7-fold), VLDL+IDL (18-fold) and LDL (14-fold) compared to C57BL/6J (C57) mice. When fed a Western-type diet, a dramatic increase in the proportions of these lipids is observed in total PC (~14-fold), particularly in the VLDL+IDL lipoprotein fraction (~30-fold) [2,4-10].

The combination of the availability of mouse models of atherosclerosis and the technology to perform hemodynamic measurements in mice has enabled the study of the effects of hypercholesterolemia and/or atherosclerosis on cardiac vascular function. In this review, we focus on studies from our laboratory and from other investigators regarding the vascular and cardiac phenotypes in the apoE^-/- ^mouse. We also discuss the underlying mechanisms that have been shown to contribute to cardiac and vascular dysfunction in this mouse model.

### Vascular phenotypes in apoe^-/- ^mice

#### Initiation and progression of atherosclerosis

The tendency to consider atherosclerosis as the effect of dyslipidemia or inflammation alone has been replaced by a new concept that this disease results from lipid disorders, enhanced oxidative stress and inflammation [11-18]. The development of atherosclerosis in conditions of hypercholesterolemia is initiated by the oxidation of lipoproteins such as LDL (oxLDL) in the subendothelial space and dysfunction of endothelial cells. In parallel endothelial cells express vascular and intercellular adhesion molecules (VCAM-1 and ICAM-1). Consequently, monocytes move into the subendothelial space where they undergo differentiation into macrophages under the influence of stimulating factors. These cells express scavenger receptors that recognize oxLDL and promote their phagocytosis. The degradation of oxLDL in lysosomes is slow, facilitating the formation of foam cells. The pivotal stage of atherogenesis seems to be antigen presentation by macrophages to T lymphocytes that recognize oxLDL and locally release proinflammatory cytokines such as interleukins (IL-1, 6, 12 and 18) and chemokines such as tumor necrosis factor alpha (TNFα) and monocyte chemoattractant protein-1 (MCP-1), thereby enhancing the inflammatory cascades. The accumulation and transformation of macrophages into large foam cells form fatty streaks that are characteristics of early atherosclerosis, which progresses into mature atherosclerotic plaques (fibrous caps) in the intima.

The mechanisms and progression of atherosclerosis in the apoE^-/- ^mice have been described before [19-22]. In this model, atherosclerotic lesions are observed throughout the macrovasculature, with the most prevalent sites located in the aortic root, aortic arch, common carotid, superior mesenteric artery and renal and pulmonary arteries. Histopathological studies of the progression of the lesions in the apoE^-/- ^mouse fed a chow diet revealed that the first signs of lesions appear at 6-8 weeks of age, as indicated by the attachment of monocytes to the endothelial cells and a disruption of the subendothelial elastic lamina [21,22]. Lesions containing foam cells and smooth muscle cells are observed at 8-10 weeks and fibrous plaques appear at 15-20 weeks [21,22]. The acceleration and severity of atherosclerotic lesions at all stages can be induced by a Western-type diet.

#### Hemodynamics: endothelial dysfunction

**Figure 1:**
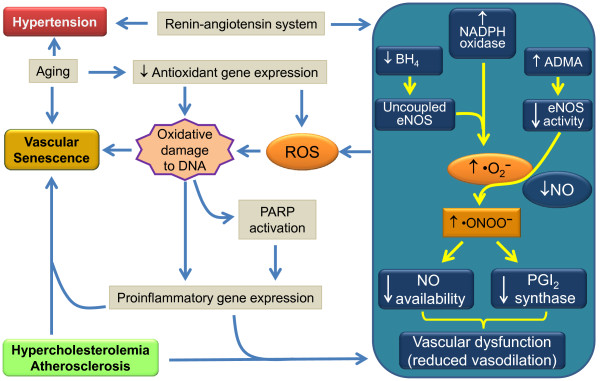
**A hypothetical scheme illustrating the role of ROS and decreased NO bioavailability in vascular dysfunction in conditions of hypercholesterolemia and atherosclerosis in the apoE^-/- ^mouse**. Abbreviations: ADMA, asymmetric dimethyl-L-arginine; BH_4_, tetrahydrobiopterin; eNOS, endothelial nitric oxide synthase; NADPH, nicotinamide adenine dinucleotide phosphate; NO, nitric oxide; •O_2_^-^, superoxide anion; •ONOO^-^, peroxynitrite; PARP, poly-ADP-ribose polymerase; PGI_2_, prostacyclin; ROS, reactive oxygen species.

Recently, we reviewed the potential mechanisms underlying endothelial dysfunction in the apoE^-/- ^mouse and how endothelial dysfunction is influenced by aging, gender and diet [23]. In brief, the conducting vessels of apoE^-/- ^mice show an impaired endothelial nitric oxide (NO)-dependent relaxation response to acetylcholine (ACh), which is associated with plaque formation [6,24,25] and aggravated by a Western-type diet [10,26-28] and by aging [24,29]. As illustrated in Figure 1, the main potential mechanism underlying the endothelial dysfunction of apoE^-/- ^mice may be the endothelial NO synthase (eNOS) pathway, due to the uncoupling of eNOS in the endothelium. This uncoupling decreases the bioavailability of NO, increases production of superoxide anion (•O_2_^-^) and generates peroxynitrite (•ONOO^-^), which is associated with the activation of the endothelin-1 (ET-1) system [6,28,30-33]. In resistance vessels, which rarely develop atherosclerotic lesions, impaired endothelial NO-dependent relaxation responses to ACh have also been observed, primarily in female apoE^-/- ^mice [34,35]. This effect appears to be mediated by hydrogen peroxide (H_2_O_2_) or an endothelium-derived hyperpolarizing factor (EDHF)-like principle agent, increased NADPH oxidase-derived •O_2_^- ^and ET-1.

Local oxidation of circulating lipoproteins and their incorporation within the vascular endothelium, associated with oxidative processes, plays a pivotal role in the pathogenesis of endothelial dysfunction in the apoE^-/- ^mouse [6,10]. The endothelial dysfunction promotes the entry and retention of LDL particles in the artery wall [36,37] enhancing the progression of lesions and creating a vicious cycle. This idea is corroborated by the finding that *in vitro *treatment of aortas with oxLDL mimicked the endothelial NO dysfunction that was observed in apoE^-/- ^mice [10]. The finding that gene transfer of human paraoxonase 1, which destroys lipid peroxides, into apoE^-/- ^mice decreased the oxLDL content of the plaques and restored endothelial function [29] corroborates this point of view. Others have shown that treatment of apoE^-/- ^mice with large empty phospholipid vesicles, which accelerates the reverse pathway of lipid transport from peripheral tissues to the liver, restored endothelium-dependent relaxation, leukocyte adherence, and endothelial expression of VCAM-1 to normal or nearly normal levels [38].

#### Hemodynamics: arterial blood pressure

Genetically engineered mouse models have been used to investigate hemodynamics in several cardiovascular diseases, including hypercholesterolemia and atherosclerosis. Several studies, in which blood pressure (BP) was recorded acutely, have shown that BP levels in young-adult (up to 30-week-old) hypercholesterolemic apoE^-/- ^mice fed a standard chow diet or a Western-type diet are similar to those of control mice [7,31,32,39-45]. Conversely, Yang et al. [46], observed normal resting values of BP in young apoE^-/- ^mice but high levels in adult apoE^-/- ^mice, compared with age-matched control mice. However, continuous and prolonged recordings of BP have revealed that apoE^-/- ^mice exhibit elevated mean 24-hour BP levels, total abolition of BP circadian cycles and increased BP variability in the very-low-frequency band, which can be improved by the restoration of vagal and NOS-mediated regulation with statins [47].

The contribution of high BP to the development of atherosclerosis, and vice-versa, is being actively debated because data involving concurrent measurements of BP and atherosclerosis in mouse models have revealed an association in some studies and a lack of association in others between hypertension and atherosclerosis [48]. One of the first studies showed that subcutaneous infusions of norepinephrine induced increases in BP and in atherosclerotic lesion size in apoE^-/- ^mice [33]. Considering that angiotensin II (Ang II) accelerates the development of atherosclerosis in both LDL receptor^-/- ^and apoE^-/- ^mice [33,49,50] and that hypercholesterolemia has been associated with activation of the renin-angiotensin system [51,52], the apoE^-/- ^mouse became an exciting model for evaluating the hemodynamic effects of Ang II in hypercholesterolemic mice (see a hypothetical mechanism in Figure 1). Currently, the suggested mechanisms by which Ang II promotes atherosclerosis include increased oxidative stress, the production of monocyte chemoattractants, and the diapedesis of monocytes [33]. Previous studies of chronic infusions of Ang II in apoE^-/- ^mice have led to three conclusions: (a) Ang II-induced atherosclerosis was not associated with an increase in BP [53]; (b) Ang II-induced hypertension was not the direct cause of the profound increase in atherosclerotic lesions [33] because this increase in BP was similar in magnitude to that observed in wild-type control mice [54]; and (c) low doses of Ang II that did not affect BP increased aortic atherosclerotic lesions [55,56]. Likewise, it has been shown that pharmacological blockade of the Ang II AT_1 _receptor in apoE^-/- ^mice or genetic disruption of the AT_1 _receptor (apoE^-/-^/AT1^-/- ^mice) reduces the inflammatory and atherosclerotic lesion process irrespective of BP [32,57]. Additionally, long-term Ang (1-7) treatment has been shown to produce vasoprotective and atheroprotective actions in the apoE^-/- ^mouse without direct effects on BP [45]. Conversely, it has been shown that AT_1 _receptor antagonist treatment lowered BP but had no effect on atherosclerosis [58]. Thus, it appears that the actions of Ang peptides on the pathogenesis of atherosclerosis and on BP occur through independent mechanisms. In our and other laboratories, this issue has been addressed by activating the renin-angiotensin system through partial clipping (stenosis) of the left renal artery (known as two kidney-one clip, or 2K1C, hypertension) in young apoE^-/- ^mice. In animals with concurrent hypercholesterolemia and activation of the endogenous renin-angiotensin system, the level of arterial hypertension was similar to that observed in normocholesterolemic wild-type C57 mice with high Ang II [41,42]. However, an increase in the thickness of the aortic wall is noted to some extent in the hypertensive C57 mouse and especially in the hypercholesterolemic apoE^-/- ^mouse, and the concurrence of both factors (hypercholesterolemia and hypertension) is observed to exert an additive effect on the aortic wall thickness in the apoE^-/- ^mouse [42]. Therefore, it appears that the development of arterial hypertension in atherosclerotic apoE^-/- ^mice is associated with other concurrent factors, particularly aging, imbalance in NOS/reactive oxygen species (ROS) production and Ang II (see schematic illustration in Figure 1).

#### Vascular remodeling

The vessel wall of a plaque-prone region expands to protect the lumen from the expanding plaque (positive remodeling) until the plaque grows so extensively that the outer lumen can no longer compensate and the plaque begins to encroach upon the lumen (negative remodeling) [59,60]. In apoE^-/- ^mice, positive remodeling has been reported in aortas at both early and late stages of atherosclerosis [42,59,61-63]. However, this observation may not apply for all large vessels as, in carotid arteries, positive remodeling has been reported at the early stages [60], but negative remodeling has been reported in aged animals [59,60]. A possible explanation for this discrepancy is that an excessive accumulation of lipids along the internal elastic lamina and within the media results in the death of medial cells and loss of the normal capacity to remodel in the carotid artery [59]. An additional and reasonable factor influencing or determining this difference between the carotid artery and the aorta in aged animals is that the animals were fed a regular diet in the study reporting positive remodeling [60], whereas the animals were fed a Western-type diet in the study reporting negative remodeling [59]. In addition to the lack of compensatory remodeling in the carotid arteries [59], we have observed that aged apoE^-/- ^mice show an inward (negative) remodeling in the coronary arteries (unpublished data).

#### Interplay among lipid and lipoprotein oxidation, DNA damage and inflammation

Oxidative stress and inflammation are intimately linked to the evolution of atherosclerosis. Oxidative stress originated primarily from ROS in mitochondria can damage cells or components of cells by initiating chemical chain reactions, such as lipid peroxidation, leading to the formation of oxLDL and oxidizing proteins or DNA [64,65]. LDL is oxidized in a slow process upon contact with ROS in the sub-intimal space by all major cells of the arterial wall via different mechanisms. ROS induce the oxidation of cholesterol and polyunsaturated fatty acids in LDL. Consequently, lipid peroxidation results in the formation of several products, such as oxysterols and aldehydes, which react with lysine residues of apolipoprotein B (apoB), generating oxLDL [14]. In addition to their metabolic deviation, oxLDL exhibits a large variety of biological and atherogenic properties involved in the activation of inflammatory, mitogenic or proapoptotic pathways, contributing to the progression of atherosclerosis [14,66].

Another cellular target of ROS is DNA; oxidative DNA lesions have been described ranging from base modifications to single- and double-strand breaks [67]. Consequently, excessive oxidative stress-induced DNA damage, which was originally described as the limited ability of a cell to divide, also appears to contribute to the pathogenesis of age-associated vascular disorders [65,68]. Recent studies in our laboratory [61] revealed vascular senescence in the aortas of atherosclerotic aged apoE^-/- ^mice but not in non-atherosclerotic aged wild-type C57 mice. This result suggests that in the aorta the occurrence of vascular senescence in aging may be related to the development of vascular disease, and this phenomenon may be the cause of the endothelial dysfunction observed in this murine model. Based on the observation that the induction of senescence in human aortic endothelial cells by the inhibition of telomere function results in decreased eNOS activity [69,70], studies with the atherosclerotic apoE^-/- ^mouse may provide an opportunity to evaluate and understand the interaction of vascular endothelial dysfunction and vascular senescence. We postulated that the renin-angiotensin system plays a dual role, contributing additively with ROS to DNA damage and additively with aging to arterial hypertension.

Oxidative damage to DNA in atherosclerosis also modifies other biochemical and regulatory pathways such as the overexpression of poly(ADP-ribose) polymerase (PARP-1) [71,72]. PARP-1 is a nuclear enzyme that recognizes and is activated by DNA strand breaks and is thus involved in DNA damage response pathways; simultaneously, PARP-1 may induce cell dysfunction or necrosis by depleting cellular energy pools [73]. Pacher et al. [74] reported that the activation of PARP-1 is associated with hypertension and aging but not with atherosclerosis. In contrast, other investigators [71,75] have shown that functional alterations in the endothelium of the apoE^-/- ^mouse are dependent on the activation of PARP-1 in endothelial cells. This activation leads to proinflammatory gene expression and thus contributes to the vascular senescence and endothelial dysfunction that are observed in atherosclerosis. Moreover, pharmacological blockade and genetic manipulations have demonstrated that endogenous PARP-1 is required for atherogenesis in the apoE^-/- ^mouse and functions by increasing the expression of adhesion molecules upon endothelial activation, enhancing inflammation, and inducing features of plaque vulnerability. Thus, inhibition of PARP1 may represent a promising therapeutic target in atherosclerosis [73]. Figure 1 illustrates the interplay among ROS, oxidative damage to DNA, the renin-angiotensin system, vascular senescence and atherosclerosis in the apoE^-/- ^mouse.

Atherosclerosis is a chronic disease, and whether it is initiated and/or aggravated by dyslipidemia depends on the contribution of oxidative stress and proinflammatory mediators. Thus, the impact of these contributors on the development of atherosclerotic lesions may reveal new treatment options, independent of serum lipids reduction. Recent studies in apoE^-/- ^mice have demonstrated a reduction in atherosclerosis development using an immunodeficiency strategy [20], low dose of the phytochemical tetrahydrocannabinol [76], antioxidant N-Acetylcysteine [77], PARP inhibition [73] or mononuclear cell therapy [63].

In relation to cell therapy, there is consistent evidence [78] that the atherosclerotic process initiated by endothelial death results in the subsequent replacement of the affected cells by endothelial progenitor cells; in addition, the evidence indicates that the cellular repair of ongoing vascular injury is mediated by progenitor cells. Indeed, treatment with spleen-derived mononuclear cells increases vascular NOS activity and restores endothelium-dependent relaxation in the aortas of apoE^-/- ^mice [79]. Moreover, mononuclear cell therapy in apoE^-/- ^mice results in the homing of endothelial progenitor cells, a decrease in oxidative stress and an upregulation of eNOS protein expression [63], which indicates that cell therapy is a promising tool for the restoration of endothelial function and the prevention of atherosclerosis development, independent of a reduction in serum lipids.

### Cardiac phenotypes in apoe^-/- ^mice

#### Resting HR

The mouse has a high resting heart rate (HR), which is related to its high metabolism. Hemodynamic measurements have shown that the resting HR in the conscious wild-type C57 mouse ranges from 520 to 650 bpm [42,80,81]. Similar values have been observed with acute measurements in conscious apoE^-/- ^mice [32,41,42,46,80,82,83]. Interestingly, some studies have shown that HR is preserved even when the BP is high in apoE^-/- ^mice fed either a standard chow diet [46] or a Western-type diet [84]. However, others have evaluated HR by continuous recording over 24 hours and observed a significantly increased mean HR and a total abolition of its circadian cycles in the apoE^-/- ^mouse compared with wild-type control mice [47]. Moreover, apoE^-/- ^mice exhibited decreased HR variability associated with abnormal neurohumoral control of HR and a defective parasympathetic drive to the heart, which were further exacerbated under a high-fat diet regimen [47,71]. It has also been observed that chronic treatment of apoE^-/- ^mice with a statin reduced their HRs to control levels and restored their circadian variations, despite persistent elevations in PC levels [47]. Although further studies are needed to clarify the mechanisms involved in the abnormal cardiac rhythm in the apoE^-/- ^mouse, there is evidence that the imbalance in NO/•O_2_^- ^production may play a pivotal role [47,71].

#### Cardiac hypertrophy

**Figure 2:**
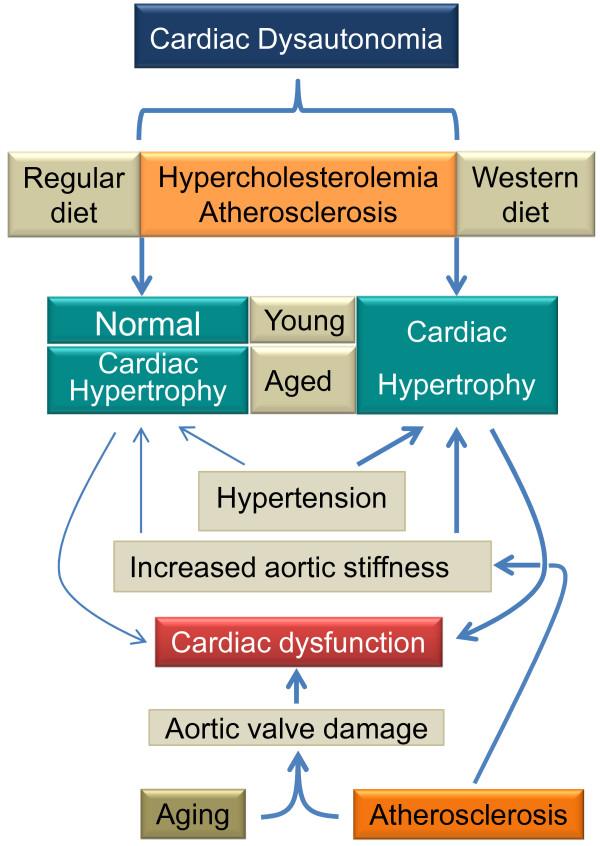
**A schematic illustration describing cardiac phenotypes and hypothetical factors that influence cardiac dysfunction in the apoE^-/- ^mouse**. Differences in line thickness indicate the relative contribution of that factor.

Cardiac hypertrophy is also an important parameter to evaluate because it can be related to cardiac dysfunction (see the illustration in Figure 2). In studies with apoE^-/- ^mice in our laboratory, we regularly evaluate cardiac hypertrophy, which can be determined by measuring the cardiac weight-to-body weight ratio [42,81]. In apoE^-/- ^mice fed a normal diet, the cardiac weight has been reported as normal at different ages in several studies [42,46,61,83,85], but others have observed myocardial hypertrophy in adult [46] and aged [80,85] animals. However, there are some controversies regarding the possible factors determining cardiac hypertrophy in aged animals. For example, it has been reported that the animals were normotensive and that the cardiac hypertrophy could be related to an elevated blood velocity and wave reflections close to the heart [80]. On the contrary, others reported both hypertension and myocardial hypertrophy in aged apoE^-/- ^mice [46]. In this case, cardiac hypertrophy could be the consequence of an increased afterload, as has been observed in non-atherosclerotic hypertensive mice [42,81]. Importantly, the cardiac hypertrophy in response to the aortic coarctation-induced pressure overload is higher in apoE^-/- ^mice than in C57 mice [83], leading to the hypothesis that apolipoprotein E may play an important role in modulating cardiac hypertrophy, even in wild-type mice. On a Western-type diet, apoE^-/- ^mice show cardiac hypertrophy that is worsened with aging [86]. Thus, despite certain controversies, studies have provided important evidence that the apoE^-/- ^mouse develops cardiac hypertrophy, which appears to be influenced by several factors, including increased aortic stiffness, arterial hypertension, and a Western-type diet combined with the aging process (see the schematic illustration in Figure 2).

#### Cardiac function

Technical progress has resulted in the ability to perform arterial and cardiac catheterization; *in vivo *imaging in the mouse is then utilized, with the aim of understanding cardiac function and dysfunction. Recently, Borst et al. [1] published an extraordinary review about methods employed for the induction and analysis of myocardial infarction in mice. However, murine models of atherosclerosis have coronary arteries that are relatively resistant to the consequences of atherosclerosis and usually do not exhibit myocardial infarction or other cardinal features of human coronary heart disease or reduced lifespan [22,87]. Hemodynamic analysis in young apoE^-/- ^mice fed a standard chow diet shows normal left ventricular end diastolic pressure and systolic pressure, cardiac output, fractional shortening, and other cardiac functional parameters [87,88]. Some studies show that feeding young animals an atherogenic diet does not seem to affect cardiac function [25], and these animals rarely show occlusive coronary artery disease and myocardial infarction under this diet [89]. However, others have reported changes in the left ventricular geometry, elevated cardiac outflow velocities, decreased distensibility of the ascending aorta and increased pulse-wave velocity, even when apoE^-/- ^mice are fed a regular chow diet [80,90,91]. In aged apoE^-/- ^mice, it has been reported that increased cardiac afterload due to increased aortic stiffening appears to contribute to a reduced cardiac reserve, indicating a reduced cardiac functional reserve in this animal [92].

Interestingly, while recently measuring the diameter of the aorta through contrast angiography, we incidentally observed aortic regurgitation associated with a larger (~3-fold) valvular thickness in elderly apoE^-/- ^mice [61]. Aortic regurgitation was observed in the elderly (hypercholesterolemic and atherosclerotic) apoE^-/- ^mouse but neither in the young (hypercholesterolemic) apoE^-/- ^mouse nor in the elderly C57 control mouse (normocholesterolemic), indicating that this cardiac dysfunction is dependent on the concurrence of hypercholesterolemia and aging [61]. In that study, the authors showed that the aortic valve damage was characterized by diffuse acellularity and myxoid thickening of the spongy layer, as well as valve fibrosis with dense collagen formation in the apoE^-/- ^mouse.

Among the animal models of heart failure, the major limitation of the apoE^-/- ^mouse is the infrequency of coronary plaques and thrombosis, two common complications of human atherosclerosis, as recently reviewed [93]. Cardiac function has also been evaluated by inter-breeding apoE^-/- ^and renin-angiotensinogen transgenic (R^+^A^+^) mice; in this animal model, heart failure develops with aging, as indicated by the decreased ejection fraction and increased lung weight in aged mice [94].

## Conclusion

Since its creation two decades ago, the apoE^-/- ^mouse, which spontaneously develops hypercholesterolemia and vascular atherosclerotic lesions on a regular chow diet, has greatly contributed to the understanding of the atherosclerosis disease process. Excess production of ROS responding to internal or environmental stress triggers several signaling steps culminating with lipid oxidation, DNA damage, senescence and production of proinflammatory substances. The renin-angiotensin system also appears to play an important role, contributing additively with ROS to changes in vascular function. Long-term recordings of HR and BP show evidence that apoE^-/- ^mice exhibit hypertension and tachycardia and abolition of their circadian cycles, primarily when under the influence of aging and a Western-type diet. Studies also provide evidence of impaired cardiac function in apoE^-/- ^mice, which is related to and/or aggravated by aging and a Western-type diet. Therefore, the studies in apoE^-/- ^mice have contributed new insights into the mechanisms underlying the cardiac and vascular dysfunction that occur in this chronic inflammatory disease.

## Competing interests

The authors declare that they have no competing interests.

## Authors' contributions

ECV, VAP, ALG, TMCP and SSM equally conceived and prepared this review. All of the authors read and approved the final manuscript.
